# Development and characterization of a topical gel, containing lavender (*Lavandula angustifolia*) oil loaded solid lipid nanoparticles

**DOI:** 10.1186/s12906-024-04440-2

**Published:** 2024-04-08

**Authors:** Faeze Fahimnia, Mehran Nemattalab, Zahra Hesari

**Affiliations:** 1 Department of Pharmaceutics, School of Pharmacy, Guilan University of Medical Sciences, Rasht, Iran; 2 Department of Microbiology, School of Medicine, Guilan University of Medical Sciences, Rasht, Iran

**Keywords:** Topical gel, Lavender oil, Solid lipid nanoparticle, Antibacterial, Wound healing, Integrative medicine, Traditional persian medicine

## Abstract

Gels loaded with nanocarriers offer interesting ways to create novel therapeutic approaches by fusing the benefits of gel and nanotechnology. Clinical studies indicate that lavender oil (Lav-O) has a positive impact on accelerating wound healing properly based on its antimicrobial and anti-inflammatory effects. Initially Lav-O loaded Solid Lipid Nanoparticles (Lav-SLN) were prepared incorporating cholesterol and lecithin natural lipids and prepared SLNs were characterized. Next, a 3% SLN containing topical gel (Lav-SLN-G) was formulated using Carbopol 940. Both Lav-SLN and Lav-SLN-G were assessed in terms antibacterial effects against *S. aureus*. Lav-SLNs revealed a particle size of 19.24 nm, zeta potential of -21.6 mv and EE% of 75.46%. Formulated topical gel presented an acceptable pH and texture properties. Minimum Inhibitory/Bactericidal Concentration (MIC/MBC) against *S. aureus* for LAv-O, Lav-SLN and Lav-SLN-G were 0.12 and 0.24 mgml^− 1^, 0.05 and 0.19 mgml^− 1^ and 0.045, 0.09 mgml^− 1^, respectively. Therefore, SLN can be considered as an antimicrobial potentiating nano-carrier for delivery of Lav-O as an antimicrobial and anti-inflammatory agent in topical gel.

## Introduction

One of the most complex organs in our body is skin which plays a serious role as a barrier against harmful microorganisms [1]. After cutaneous damages or chronic bedridden conditions invading bacteria may create a biofilm on the surface of the injured skin and enter the subcutaneous tissues, which may lead to impaired wound healing and fatal infections [2–4]. Due to the microbiological complications they bring about, such as local or apparent infection, slow healing, and the emergence of multi-resistant bacteria, wounds are a major public health concern in developed countries [5].

Despite being a normal component of human skin and nasal microflora, *Staphylococcus aureus* (*S. aureus*) is one of the foremost opportunistic bacterial pathogens of humans, and one of the multi-drug resistant pathogens that causes several pathogenic conditions, such as bacteremia, necrotizing pneumonia, skin and soft tissue infections, *S. aureus*-induced surgical-site infection [6–10]. Furthermore, finding new therapeutic choices for this pathogen is urgently required due to the rise and spread of antibiotic resistance, particularly methicillin-resistant and vancomycin-resistant *S. aureus* (MRSA and VRSA, respectively) [11–13]. On the other hand, most Gram-negative and Gram-positive bacteria, including *S. aureus* have the ability to form biofilms [14] which are a survival strategy of bacteria that allow them to escape the host defense mechanisms and cause recurrent chronic infections [15, 16].

Several essential oils (EOs) of herbal origin contain numerous compounds with known antimicrobial activity and broad mechanisms of action [17–19]. Lavender (Lavandula angustifolia) is one of the most precious plants from the range of medicinal and aromatic plants with commercial importance and it is grown for industrial purposes [20]. Lavender oil (Lav-O) possesses sedative, carminative, antidepressant, and anti-inflammatory properties in addition to its antibacterial properties [21]. Clinical studies indicate that Lav-O has a positive impact on how quickly wounds heal. Additionally, it was noted that both animal trials and human studies using Lav-O as a topical treatment for aphthous ulceration revealed a substantial decrease in ulcer size when compared to controls [22]. Besides, lavender essential oils have already been explained as a wound-healing agent due to their capacity to accelerate wound contraction [23]. .

Gels that have been loaded with nanocarriers offer interesting ways to create novel therapeutic approaches by fusing the benefits of gel and nanotechnology [24]. Topical use of gels at pathological locations provides substantial advantages over creams and ointments due to their high amount of water, allow a greater drug dissolution. Also, gels provide skin hydration by retaining a noticeable trans-epidermal water content and facilitate drug absorption [25, 26]. The three-dimensional polymer network of the gel offers an ideal opportunity for the prolonged release of therapeutic molecules, such as proteins, improved mucoadhesive properties in the regeneration of bone tissues, the elimination of exudates, the wound healing due to its adjustable viscosity, and gel-based techniques are being developed to improve their administration using stimuli-responsive in situ gelation [27, 28]. Due to their hydrophilic properties, the cross-linked structure of Carbopol is a potential candidate to use as a gel-like formulation for current application in transdermal drug delivery [29].

Solid Lipid Nanoparticles (SLNs) are physiologically well tolerated due to the matrix consisting of solid lipids, and the low cytotoxicity of this system, also these nanoparticles show high potential as a suitable drug delivery system [30, 31]. Among the advantages of solid lipid systems are the potential of combination of lipophilic and hydrophilic drugs, physical stability, controlled drug release, biocompatibility, increased bioavailability of the drugs, site-specific drug delivery, improved drug stability, enhanced pharmacokinetic profile of the drugs, high drug loading, controllable particle size, and easy scale-up and fabrication are noted [32]. In addition, SLNs have increased dermal penetration, occlusive characteristics, and a longer residence time inside the skin layers, making them an efficient dermal drug delivery route of administration. Also, these carriers are compatible for use on inflamed skin as well, because of the non-skin irritating character of the lipid matrix. It has been demonstrated that the incorporation of SLNs into gels increases drug permeability in comparison to conventional gel formulations. This has been explained by the interaction of SLNs with the stratum corneum, which leads to a longer residence time in skin layers [33, 34].

However, there are various studies on fabrication of nano-delivery systems (nanoparticles, nanofibers, etc.) of lavender oil, to our knowledge, it is the first study incorporating only natural lipids (cholesterol and lecithin) for SLN synthesis. Also, in this study, a simple approach was adopted for the synthesis of lavender oil-loaded solid lipid nanoparticles (Lav-SLN). Therefore, we attempted to develop a transdermal Carbopol gel containing Lav-SLN (Lav-SLN-G). After preparation and physicochemical characterization, we tested the antibacterial activity of the Lav-SLN-G against *S. aureus*.

## Materials and methods

Cholesterol (Sigma, Germany), soya lecithin (DUKSAN reagents, South Korea), PVA (Merck, Darmstadt, Germany) (800,000 Da), tween 80, dichloromethane and methanol (all with analytical grade; Merck, Germany), carbomer 940 (Lubrizol Pharmaceuticals), Mueller Hinton Broth/Agar (MHB/A) (Merck, Germany) were purchased.

### Preparation of Lav-SLNs

In this research, SLNs containing Lav-O were prepared by emulsification ultrasonic-homogenization method. Tween 80 was utilized as a surfactant, also lecithin and cholesterol were used as lipids. Briefly, 0.8 ml Tween 80, 100 mg lecithin, and 100 mg cholesterol along with 1 ml of Lav-O were dissolved in 10 ml dichloromethane. Then, 10 ml of PVA 4% solution was added to aforementioned mixture and homogenized for 10 min at 15,000 rpm using an ultrasound probe sonicator (Hielscher UP400s, Germany) to produce a cloudy white emulsion. The organic solvent was entirely evaporated from the final w/o/w using a rotary evaporator at 35 °C for 20 min [35].

### Characterization of Lav-SLN

#### Particle size, polydispersity index (PDI) measurement, and Zeta potential

The zeta potential and particle size of Lav-SLN were evaluated using a Zetasizer (Malvern Instrument, UK). 0.015 ml of the nanoparticles were suspended in 1 ml of deionized water and the average particle size was determined by Zetasizer Ver. 6.01 software at 25 °C with a count rate of 206.3 kcps and measurement position of 4.65 mm. For the evaluation of the size distribution (monodisperse or polydisperse nature) of nanoparticles, the polydispersity index was calculated. The higher polydispersity index values (≥ 0.7) illustrate a high level of non-uniformity [36, 37].

#### TEM analysis

The size and morphology of nanoparticles were determined by a TEM microscope (Zeiss-EM10C-100 KV, Germany). In this procedure, the nanoparticle suspension was placed in drops on the mesh grids coated with a Formvar. Subsequently, 10 min dehydration for each of the following ethanol concentrations (50%, 70%, 90%) was performed; the last dehydration was performed in 100% EtOH. After dehydration, the ethanol was removed and the samples were dried at 37 °C. Then examined using a Transmission electron microscope at a voltage of 80 kV.

#### FTIR spectrometry

Lav-O and Lav-SLN were analyzed using an FTIR spectrometer (PerkinElmer ES Version 10.5.3, USA) to investigate potential incompatibilities between Lav and added excipients. FTIR Spectra were collected at a resolution of 4 cm^− 1^and given as the ratio of 21 single beam scans to the same number of background scans in pure KBr [38].

#### % entrapment efficiency (EE)

In various studies, the EE of nanoparticles or microspheres was generally determined in an indirect way in which the amount of drug entrapped in nanoparticles was determined by the difference between the total amounts of drug (W_1_) and the unentrapped drug after centrifugation (W_2_) [39]. To estimate the quantity of unentrapped Lav-oil in the supernatant fluid (W2), initially, 10 mL of distilled water containing 5% Tween 80 was used to disperse 500 mg of Lav-SLN. The aqueous dispersion was centrifuged at 10,000 rpm for 40 min at 4 °C. Utilizing UV–vis spectrophotometry (Agilent Technologies, Cary 60, Santa Clara, CA, USA) at 228 nm, the amount of unentrapped Lav-oil in the supernatant fluid was measured and percentage of entrapped oil in nanoparticles was estimated as follows [40]:

EE (%) = (W_1_ - W _2_) / W_1_ × 100.

W_1_ = Total amount of Lavender oil.

W_2_ = quantity of un-entrapped Lavender oil (free).

### Preparation of gel containing Lav-SLN (Lav-SLN-G)

Carbomer 940 (0.5% w/v) was used as a gelling agent to formulate Lav-SLN-G after optimizing the selected variables. 0.5% of Carbopol was dispersed in water for 24 h to prevent the formation of lumps. After 24 h, Lav- SLN dispersion equal to 3% in gel’s final volume was added and completely uniformed using a mechanical stirrer. Next, 20% propylene glycol and 3–4 drops of triethanolamine were added to the product, respectively. As soon as triethanolamine was added, an enhanced viscous solution turning to gel was observed [41].

### Physicochemical characterization of gel

The SLN dispersion in gel was examined for morphology, surface tension, viscosity, pH, and spreadability to determine their suitability.

#### Morphological analysis

Morphology of nanoparticle dispersions in polymer media was investigated by FESEM Sigma VP (Zeiss, Germany) equipped with an energy-dispersive X-ray spectroscopy system with an Oxford INCA detector. For sample preparation, surface of a glass slide was placed in contact with Lav-SLN-G containing 3% SLN leading to attachment of a gel layer on slide surface. This gel layer was air dried at 25 °C in a desiccator and dried gels were gold sputter coated before FESEM. Samples were observed using an accelerating voltage of 10 kV, a working distance of around 4.4 mm, and magnification between 1 and 50 KX [42].

#### Texture analysis

The springiness, hardness, gumminess, chewiness and cohesiveness of Lav-SLN-G containing 3% SLN were analyzed using a Texture Analyzer (Brookfield CT3 Texture Analyzer, Brookfield Engineering Laboratories). A 25.4 mm cylindrical probe was used for the test at 25 °C [43].

#### Viscosity

The viscosity of the formulated gel was determined using a Brookfield viscometer (Model RV – DV I Prime, Brookfield Engineering, MA, USA). Before measuring, the sample was spinned with a RV spindle at 50 rpm and was given five minutes to acclimate. The measurements were repeated in triplicate at 22 °C [44].

#### Determination of pH and spreadability

The pH of the prepared gel was determined using a digital pH meter (Model Proline B 210, China). The spreadability (S) of the gel was assessed by placing 100 mg of the sample between two glass slides. Then a weight of 20 g was added to the upper glass slide and the time of separation of the upper slide (movable) from the fixed slide was determined, and spreadability was calculated using the formula [45, 46].

S = ML/T.

where:

S = Spreadability of gel,

M = Weight (g) applied on the upper slide,

L = Length (cm) of the glass slides,

T = Time taken to separate the slide completely from each other.

### In-vitro release and release kinetic study

The *in-vitro* release test was performed for Lav-SLN and Lav-SLN-G separately. The dialysis bag with a molecular weight cutoff of 14 kDa (Sigma, Steinheim, Germany) was filled with 1.2 g of nanoparticles and 2 g of Lav-SLN-G and sealed on both sides. The bag was immersed into the 100 mL distilled water containing 5% v/v tween 80 as receptor medium, at 32 °C with constant stirring (100 rpm) [47]. 3 ml of samples were collected at various time points of 0, 1, 6, 24, 48, and 72 h and substituted with fresh medium to maintain the sink condition. Then, analyses of the Lav-O content were carried out using UV-Visible spectrophotometry at a wavelength of 228 nm [48].

To explore the release kinetics of Lav-SLN and Lav-SLN-G, the in vitro release data was matched with mathematical kinetic models of zero order, first order, Higuchi, Korsmeyer-Peppas, and Hixson-Crowell [49].

### Cell compatibility assay

Using the MTT assay, the antiproliferative effects of the Lav-SLN on the HU02 (Foreskin fibroblast) obtained from Iranian Biological Resource Center (IRIBC C10309), cell lines were assessed. Briefly, cells were cultured for 24 h in a 96-well plate with a density of 5×l0^3^ cells/cm^2^ in 0.1 ml of DMEM medium in a humid environment with a 5% CO_2_. The samples of Lav-SLN (50 mgml^− 1^) and Lav-O (25, 50 mgml^− 1^) containing 5% v/v tween 80 media were applied to the cells. Following a 24 h treatment period, the cells were treated with MTT solution (Sigma; 5 mgml^− 1^ of PBS) for 3 h at 37ºC. The medium was then taken out, and the precipitates created were dissolved in 0.15 ml of DMSO for each well. Subsequently, using a Biotek EpochTM microplate reader at 570 nm, absorbance was measured (*n* = 3) [50].

### Antibacterial assay

#### Bacterial strains and culture conditions

In this study, the antimicrobial activity of Lav-O, Lav-SLN, and Lav-SLN-G were investigated against the standard sample *S. aureus* (ATCC 25,923). Bacterial standard stock kept at -30 °C was cultured on freshly prepared BHI agar plates and incubated aerobically at 37 °C for 24 h.

#### Minimum inhibitory concentration (mic) and minimum bactericidal concentration (mbc) determination

The broth microdilution method was used to calculate the MIC and MBC. Test strain standard cell suspensions were created utilizing 24 h bacterial cultures. On a 96-well sterile plate, the Lav-O, Lav-SLN, and Lav-SLN-G solutions were serially diluted with sterile MHB. Each well’s final microbe concentration was set at 10^6^ CFUml^− 1^. Plates were then incubated for 24 h at 37 °C and plates were visually inspected for turbidity. The lowest concentration of additives that prevented bacterial growth was identified as the MIC values. To ensure accuracy of the evaluation of bacterial growth inhibition in gel series, a blank gel series with no bacteria was considered as control and the absorbance of each well was determined using an ELISA reader with a wavelength of 640 nm. To determine the MBC, 0.1 mL of no growth well solutions were cultured on MHA plates and incubated at 37 °C for another 24 h aerobically. The lowest concentration of samples required to completely kill the bacterial population was defined as MBC [51, 52].

#### Determination of inhibition zone

To measure the inhibition zone of Lav-O, Lav-SLN and Lav-SLN-G against *S. aureus* (ATCC 25,923), equal concentrations of all three samples (5 mgmL^− 1^) were subjected to MHA wells at 32 ± 2 °C for 48 h.

### Statistical analysis

Graphpad prism 8.0 software and SPSS version 22.0 was used to process data for the statistical analysis. Multiple means were compared using one-way and ANOVA tests. The level of significance was taken at 5% (*p* < 0.05).

## Result

### Characterization of Lav-SLN

#### Particle size, zeta potential and TEM analysis

**Fig. 1:**
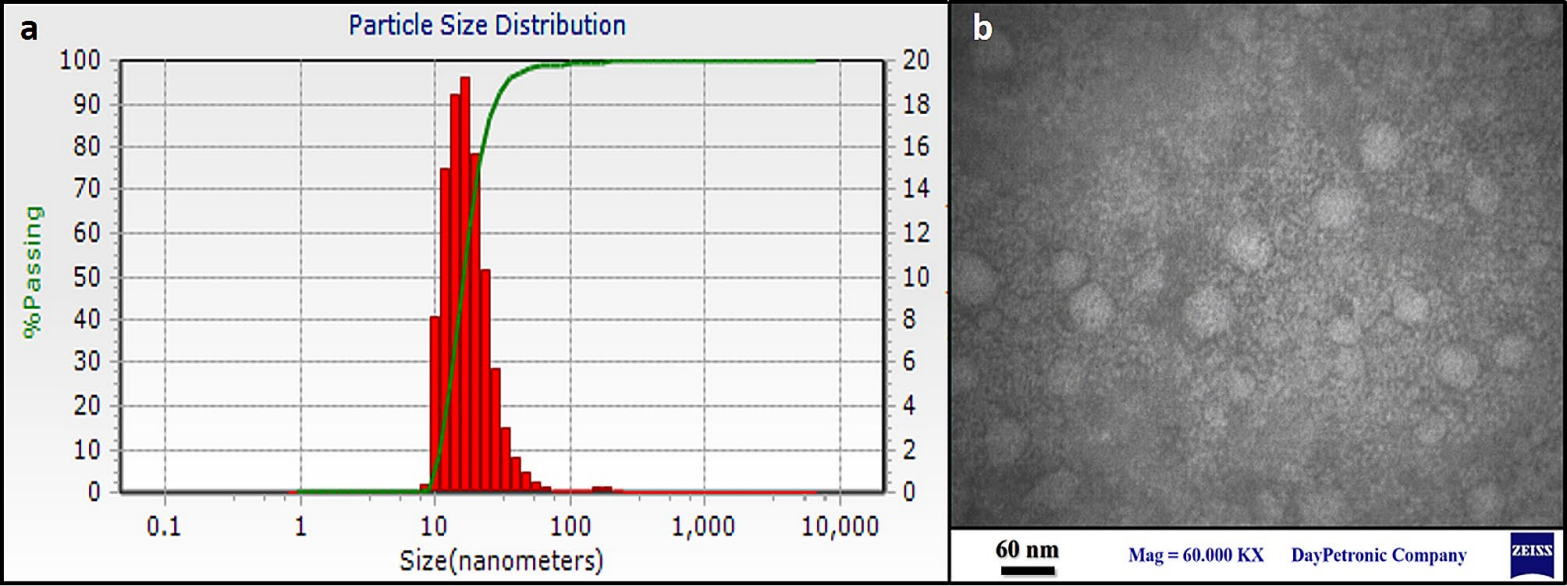
Lav-SLN characterization; (**a**) particle size analysis by DLS. (**b**) TEM two-dimensional morphological description

The prepared solution of Lav-SLN nanoparticles presented an average size of approximately 19.24 nm and with a particle size distribution (PDI) of 0.079 (Fig. 1a). Also, the zeta potential of the nanoparticles was measured at − 21.6 mV revealing a relatively high zeta potential results in stronger electrostatic repulsion which prevents particle aggregation and leads to better size stability. Also, the two-dimensional morphological information of SLNs containing Lav-O is shown in Fig. 1b. In these pictures, TEM micrographs confirmed a uniform round shape with smooth surface particles for Lav-SLN.

#### FTIR spectrometry

**Fig. 2:**
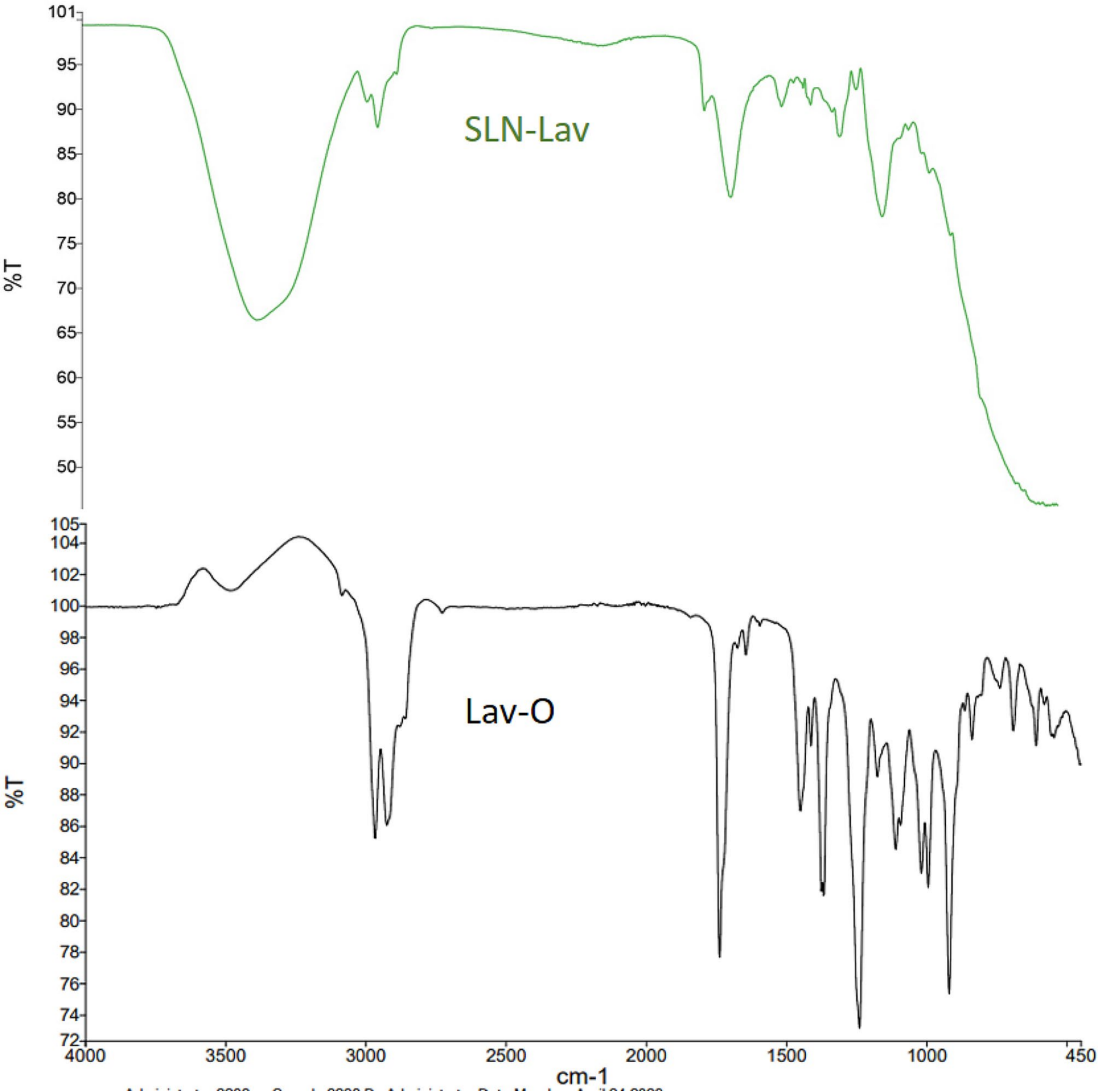
FTIR Spectra of Lav-O in black along with Lav-SLN in green

The FTIR spectra of Lav-O and SLN containing Lav-O are presented in Fig. 2 to confirm the stability of Lav-O during SLN preparation. Worth mentioning, the spectra of tween 80, lecithin and cholesterol were previously published and discussed [51].

#### Entrapment efficiency (EE)

The percentage of incorporated Lav-O in the lipid matrix was evaluated. The incorporation of Lav led to high entrapment efficiency (75.46%), probably due to its lipophilic character.

### Characterization of Lav-SLN-G

#### Evaluation of Lav-SLN-G physical appearance

The formulated gel was checked visually for color, appearance, skin feel, smell, and homogeneity; The Lav-SLN-based gel had a homogenous composition with milky color, and absence of aggregates with a mild fragrance.

#### SEM analysis

**Fig. 3:**
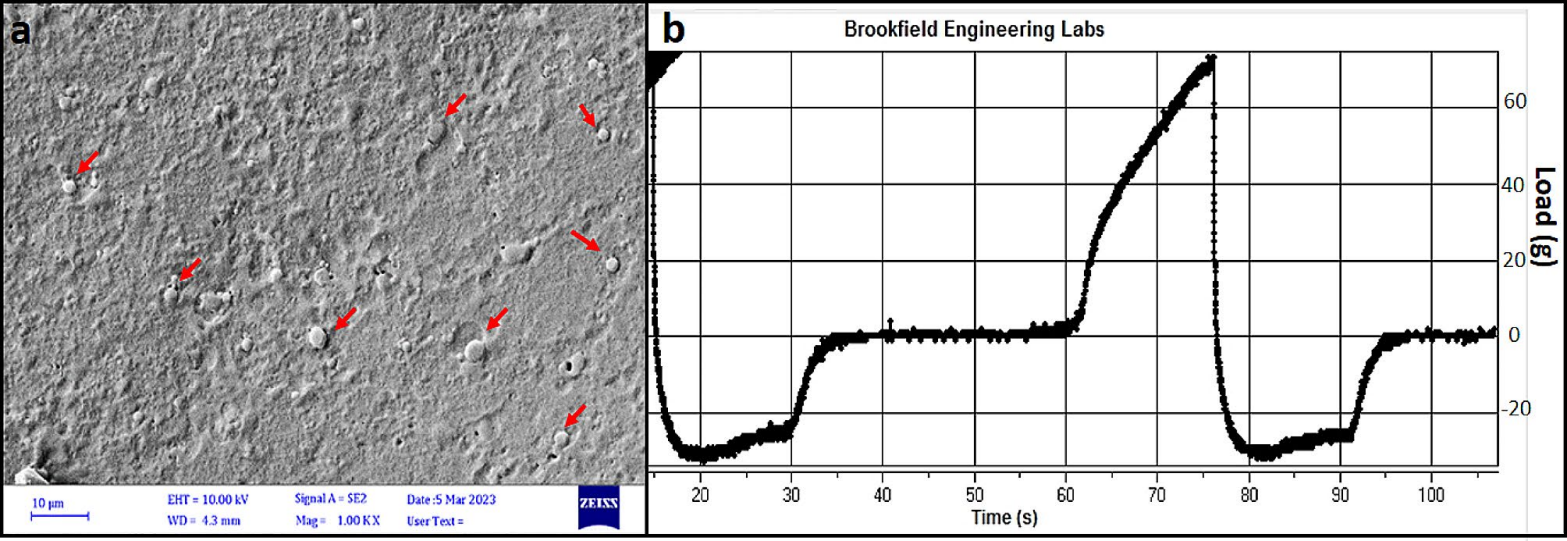
**a**) SEM image of Lav-SLN dispersed in polymeric gel matrix. **b**) Texture analysis of LAV-SLN-G containing 3% nanoparticles

SEM is a typical technique for evaluating surface morphology and nano-formulation properties. The scanning electron microphotograph of the gel formulations revealed that LAV-SLN-G was uniformly formed and had a well-defined perimeter (Fig. 3a). In addition, there was no visible agglomeration of the lipid nanoparticles in the SEM images, indicating a relatively consistent dispersion of the formulation which are pointed in Fig. 3a.

#### pH determination and spreadability

In this study, the pH of the resulting formulations was measured using a pH meter during each step of preparing the gel containing Lav-SLNs. The results obtained from formula 5.54 are reported. The pH of the prepared gel showed its compatibility with the skin. The spreadability of the Lav-SLN-G formulation was studied. The value of spreadability was determined to be 18.51 g.cm/sec, indicating that the gel could be spread easily with minimal shear. Even with a blank gel, the same findings were obtained.

#### Determination of viscosity

The viscosity of the prepared gel formulation was evaluated at a temperature of 22 degrees Celsius at a speed of 50 rpm. A result of this test was 7476 CPs were reported.

#### Texture analysis

A sample of gel, containing 3% Lav-SLN was subjected to texture evaluation at 25 °C. The behavior is shown in Fig. 3b and springiness, hardness, gumminess, chewiness and cohesiveness amounts are presented in Table 1. According the observed values of hardness, cohesiveness and springiness, the gel provides a reliable consistency while considering the gumminess and chewiness values the potential of adhesion of gel to dermal/wound surfaces is also acceptable.

**Table 1:** Texture analysis parameters of LAV-SLN-G containing 3% nanoparticles

Hydrogel	Hardness (g)	Cohesiveness (g*s)	Springiness (mm)	Gumminess (g)	Chewiness(mJ)
Lav-SLN-G (5%)	**74.00**	**0.96**	**1.443**	**71.0**	**102.45**

### Ex vivo drug release and release kinetic studies

**Fig. 4:**
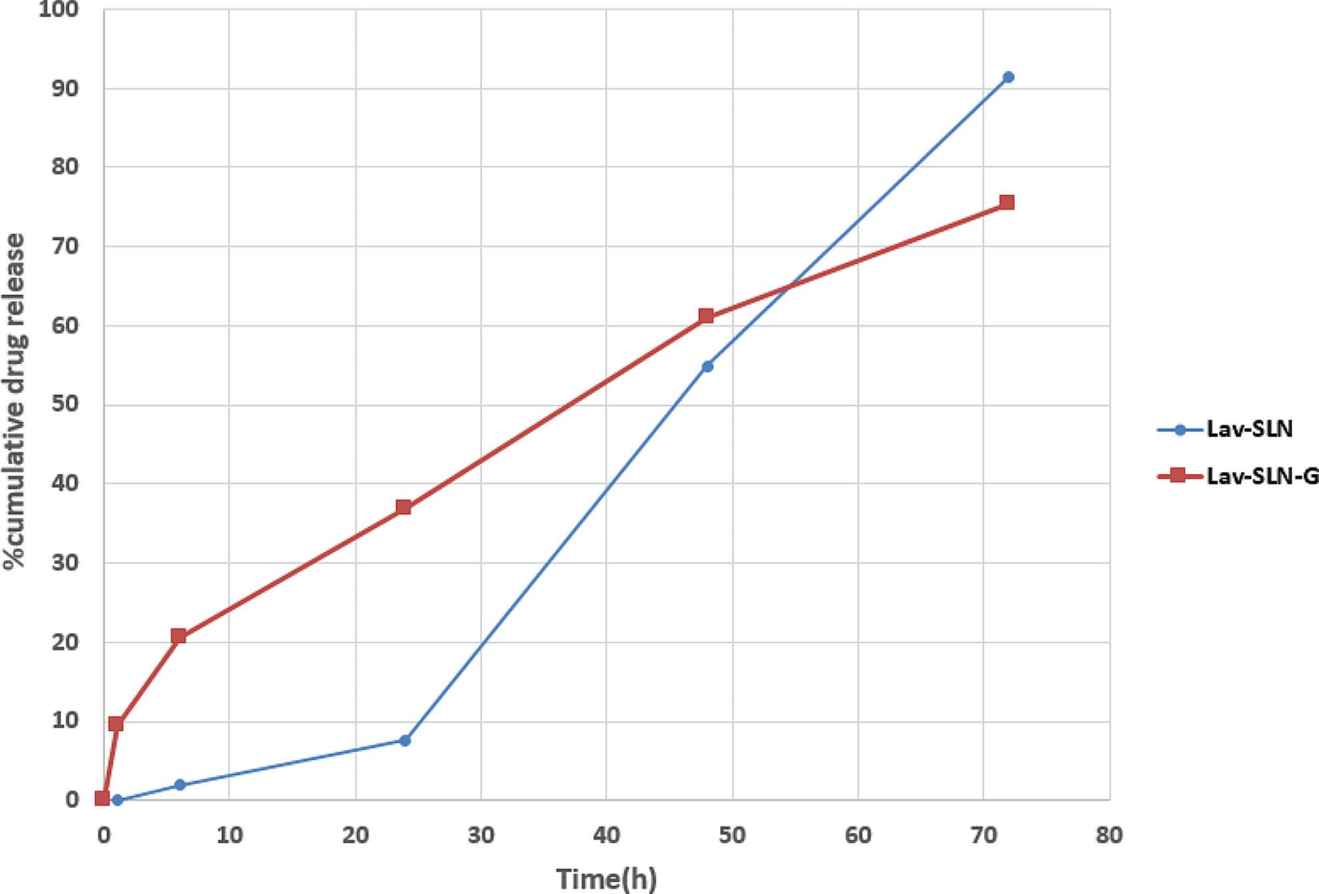
Cumulative release profile of Lav-O from Lav-SLN and Lav-SLN-G in 72 h

As is observable in Fig. 4, The cumulative oil release% in 72 h reaches to 91.44% and 75.40% from Lav-SLN and Lav-SLN-G, respectively. These results confirm that incorporation of Lav-SLN in gel matrix can enhance the sustain delivery of Lav-O on skin. Also, release kinetic calculations presented the highest regression coefficient for Zero order kinetic in Lav-SLN and First order kinetic in Lav-SLN-G (Table 2).

**Table 2:** Release kinetic parameters for Lav-SLN and Lav-SLN-G based on different mathematical models

	Zero order K_0_	Zero order R^2^	First order K_0_	First order R^2^	Higuchi K_0_	Higuchi R^2^	Hixson K_0_	Hixson R^2^	Kors-Peppas K_0_	Kors- Peppas R^2^
SLN	1.27	0.9486	-0.013	0.853	10.209	0.8028	0.0339	0.8987	37.34	0.6376
G7	0.98	0.960	-0.008	0.993	8.7377	0.9917	0.023	0.9895	34.432	0.9143

### Cell viability

**Fig. 5:**
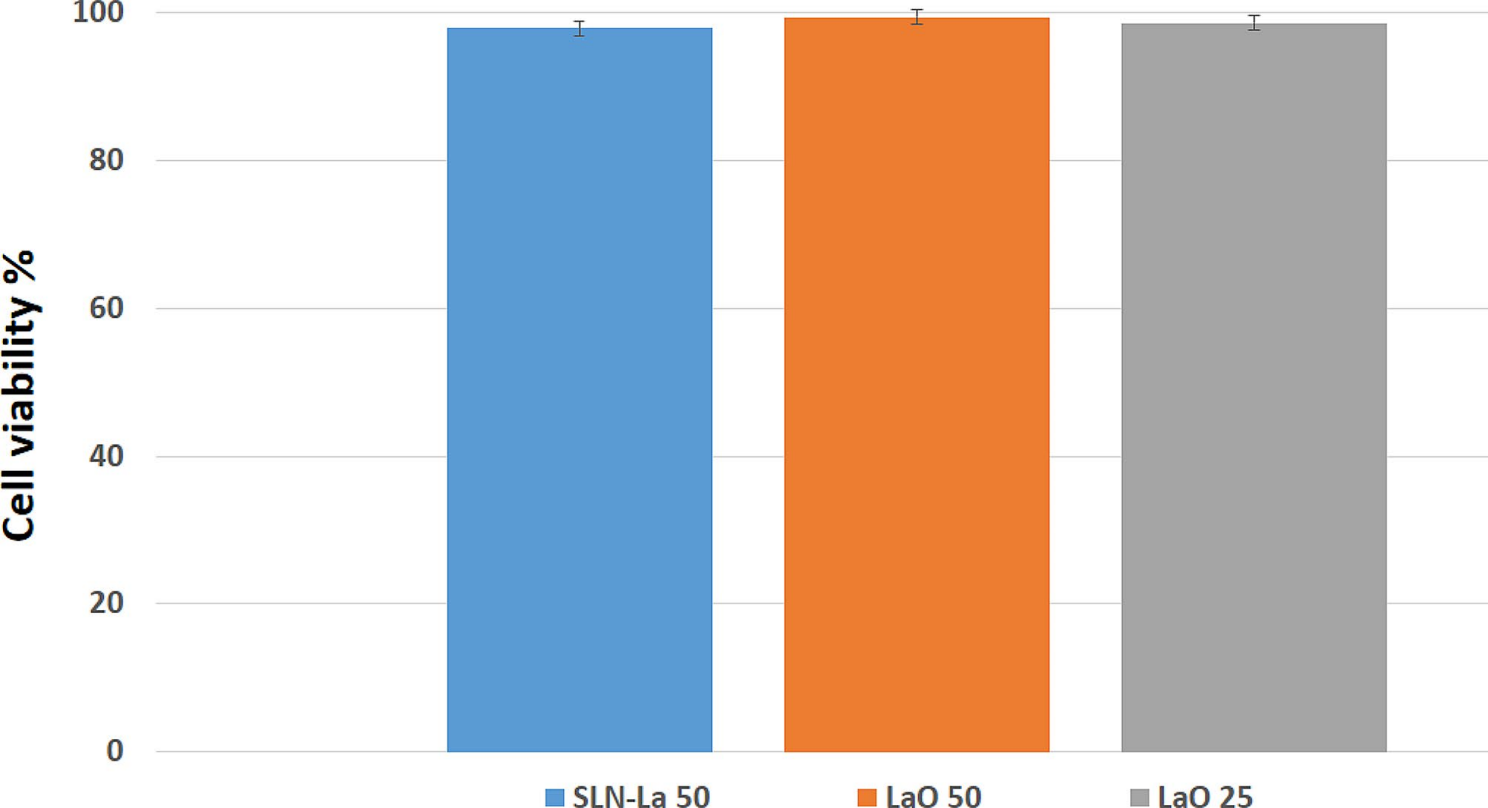
The HU02 cell line viability% exposed to Lav-O (50 and 25 mgml^− 1^) compared to Lav-SLN (50 mgml^− 1^)

Probable toxicity of the Lav-O and Lav-SLN was evaluated on HU02 (Foreskin fibroblast) cell lines using an MTT method. The Lav-O was tested with the concentrations of 25 and 50 mgml^− 1^while Lav-SLN was evaluated with the concentrations of 50 mgml^− 1^. Results revealed that there was no significant difference between the cell viability for Lav-SLN (97.96 ± 0.01%) and different concentrations of Lav-oil (98.59 ± 0.005% for LaO-25 and 99.42 ± 0.01% for LaO-50). Additionally, the cell viability for all samples was higher than 90% confirming the no toxic effects of Lav-O and its SLN (Fig. 5).

#### In vitro antimicrobial activity assessment

After 24 h of incubation, antibacterial activity was examined in all the wells containing Lav-O, Lav-SLN, and Lav-SLN-G against *S. aureus* in terms of turbidity (Table 3). The best antimicrobial activity against *S. aureus* was shown by the Lav-SLN-G with a MIC of 0.045 mgml^− 1^. The range of MIC in Lav-SLN and Lav-O was 0.05 mgml^− 1^ and 0.12 mgml^− 1^, respectively. Comparing MIC ranges reveals, SLN potentiates the antibacterial effect of Lav-O, and a significant increase is observed in the antibacterial effect of Lav-O with SLN nanoparticles as a delivery system while, the MIC of Lav-SLN was not significantly different from that of Lav-SLN-G. Next, MBC was determined for all screened isolates. The MBC values were recorded for Lav-O 0.24 mgml^− 1^, Lav-SLN 0.19 mgml^− 1^, and Lav-SLN-G 0.09 mgml^− 1^against *S. aureus* isolates. Compared with the case of Lav-O, the MBC of Lav-SLN and Lav-SLN-G were statistically significant, also Lav-SLN-G significantly reduced the amount of MBC compared to Lav-SLN and Lav-O.

**Table 3:** The MIC and MBC values (mgml^− 1^) for Lav-O, Lav-SLN and Lav-SLN-G against *S. aureus*

Lav-O		Lav-SLN		Lav-SLN-G	
MIC	MBC	MIC	MBC	MIC	MBC
0.12	0.24	0.05	0.19	0.045	0.09

#### Determination of inhibition zone

**Fig. 6:**
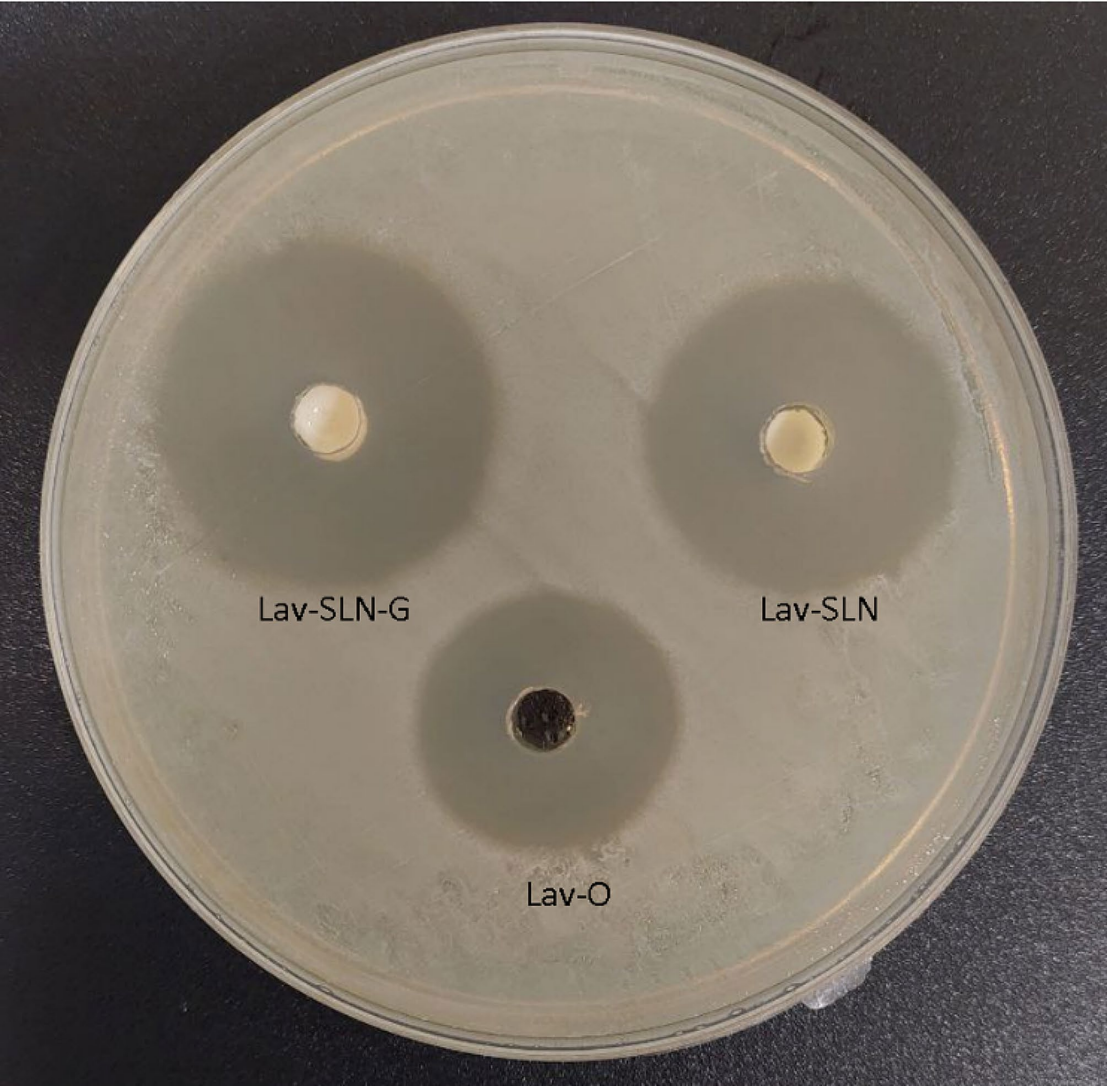
Inhibition zones of Lav-O, Lav-SLN and Lav-SLN-G (5 mgmL^− 1^) against S. aureus (ATCC 25,923) in 48 h

The inhibition zone of Lav-O, Lav-SLN and Lav-SLN-G with equal concentrations of 5 mgmL^− 1^ against *S. aureus* (ATCC 25,923), were determined and presented in Fig. 6 in 48 h.

## Discussion

The mean particle size, polydispersity index, and zeta potential of SLNs are crucial characteristics that can help us anticipate the stability and function of nanoparticles. This is due to increased steric stabilization and reduced electrostatic stabilization [41].

In current study, the hydrodynamic diameter of the Lav-SLN was reported 19.24 nm with the preparation technique of emulsification ultrasonic-homogenization while Chaudhari et al., reported the fabrication of lavender oil loaded SLN using hot homogenization technique which led to the lowest particle size of 30.91 nm [41]. It seems that both techniques are able to provide particles with the diameter below 50 nm. Also two other studies formulated Nano lipid carriers (NLC) incorporating lavender oil using Phase Inversion Temperature method and double emulsification method but the resulted nano-carriers revealed a significantly higher particle size (99.88 and 398.8 nm), respectively [53, 54]. In the study of Reeta et al., Lavender oil NLC revealed a 17.18 mV surface charge while incorporation of softisan as NLC lipid in Carbone et al., study presented a negative charge of -5.02 mV. However, it seems that formulation of SLN from lavender oil using cholesterol in current study resulted in a more stable zeta potential of -21.6 mV. The cumulative oil release% for Lav-SLN was 91.4% in 72 h while in Chaudhari et al., study Lav-O release was around 90% but in 8 h which shows a faster release compared to current study that may be related to the incorporated lipid type (cocoa butter versus cholesterol) [41]. Similarly, several other investigations have evaluated the release of drugs, loaded in SLN and dispersed in semisolid bases in which the release kinetic of drug from SLN was different with the kinetic of release from SLN dispersions in gel [55]. For example, zaltoprofen was released from SLN with Higuchi kinetic while release of the same drug from SLNs dispersed in gel, followed the Korsmeyer peppas model [56] or meloxicam released with zero order kinetic from SLN and Higuchi diffusion model from SLNs suspended in gel [57]. It seems that the type of release kinetic depends on the lipophilicity of drug, types of incorporated lipid in SLN and texture characteristics of gel matrix. Consequently, it is anticipated that the sustained release of Lavender from preparations based on Lav-SLN and Lav-SLN-G will be beneficial in reducing skin irritation and promoting prolonged activity, with improved permeation.

Many studies have fabricated lavender oil containing semisolid/liquid formulations, but, to the best of our knowledge they have mainly evaluated the viscosity or rheometric behavior of the formulations [58–61]. However, some studies have investigated the influence of essential oils and their composites on the texture of hydrogels; for instance, Wang. et al. fabricated kappa–carrageenan hydrogels (KC) containing cinnamon essential oil (CEO)/hydroxypropyl–β–cyclodextrin composite (HPCD). Textural analysis revealed that the hardness of KC gel was 252.01 g which reached to a maximum of 300.74 g with incorporation of 3% (CEO/HPCD) which is higher than the hardness of Lav-SLN-G 3% which was 74 g. In addition, the springiness of KC gel was 0.979 which slightly increased to 0.988 by addition of 3% composite that is lower compared to Lav-SLN-G 3% (1.443) revealing a less brittle and more flexible texture of Lav-SLN-G. Also, both gumminess and chewiness as the secondary parameters were reported around 106 in KC gel with 3% CEO/HPCD which were higher than our gel [62].

*Lavendula sp*. essential oils have been both therapeutically and cosmetically used for centuries. The oil is traditionally believed to have sedative, carminative (smooth muscle relaxing), anti-depressive and anti-inflammatory properties, effective for burns and insect bites in addition to its recognized antimicrobial effects [63, 64]. Also, its wound healing effects have been widely claimed and studied which may be partly related to its antimicrobial effects; since, antimicrobial action is of great focus in wound dressing [65, 66]. Hence several studies have evaluated and confirmed the antibacterial characteristic of Lav-O alone or in combination with other herbal derivatives [67–69].

For the production of commercial products based on Lav-O, overcoming some limitations such as chemical instability in the presence of air, light, moisture, and high temperatures [70] is essential. Various encapsulation techniques have been utilized as a practical tool in this regard [21, 71]. In addition, incorporation of nano-carriers has been confirmed to enhance the skin penetration of loaded drugs [72, 73], especially lipid carriers [74, 75]. Hence, a Lav-O topical gel was designed for potential application in wound healing, while encapsulating Lav-O in SLN carrier.

After encapsulation, FTIR technique is used to investigate probable instability or incompatibilities of the entrapped oil. For Lavender oil, the absorption bands at 3445.21 cm^− 1^, indicates the (-OH) group. The comparable C-H stretching vibration for methylene groups are found near 2965 cm^− 1^ and for methyl groups near 2922 and 2872 cm^− 1^; along with bending vibration for methylene in 1465 and for methyl in 1375 cm^− 1^. The strong bands related to C = O (1727 cm^− 1^) and C–O (1253 cm^− 1^) indicated the stretching of ester groups. All mentioned peaks are in accordance with previously published Lav-O spectra [76, 77]. While in Lav-SLN spectrum, the C-H stretching peaks 2965 − 2872 cm^− 1^, C = O doublet peak on 1727 and C-H bending peak on 1375 cm^− 1^ are all matched with Lav-O spectrum confirming the compatibility of the oil with SLN excipients.

However, the antibacterial effects of a gel containing Lav-SLN was not evaluated yet, several studies have been investigated the influence of nano-delivery systems on Lav-O antimicrobial effects [78], as follows; D. Predoi., et al. reported the MIC of 0.2 mgml^− 1^ for Lav-O hydroxyapatite nanoparticles (Hap-L) [79] against *S.aureus*. Also, in another study the MIC of Lav-O against *S.aureus* decreased from 1.2 mgml^− 1^ to 0.45 mgml^− 1^ when it was nano encapsulated in hydroxypropyl-beta-cyclodextrin [80]. S. Das., et al. presented the MIC of Lav-oil encapsulated in randomly methylated β cyclodextrin was 1.25–2.5 mg/ml against *S.aureus* which is in a significantly higher range compared to our results [81]. In addition to nanocapsules, Lav-O and its antimicrobial properties have been incorporated in nanofibers. For example, polyacrylonitrile (PAN) nanofibers containing 0.1 mgml^− 1^ Lav-O (in polymeric solution) presented a 14–15 mm inhibition zone diameter against both *S.aureus* and *K. pneumonia* [76]. Also, Lav-O was incorporated in polyurethane nanofibers along with silver nanoparticles which revealed an inhibition zone of about 6 mm against *S.aureus* with the concentrations of 15% Lav-O and 5% Ag NPs [82]. While, in current study, MIC for Lav-O was 0.12 mgml^− 1^ which decreased significantly in SLN form to 0.05 mgml^− 1^ and remained its activity in gel formulation with the MIC of 0.045 mgml^− 1^ (decrease in gel, was not statistically significant). As is observable, SLN carrier have notably potentiated the antibacterial properties of Lav-O for a longer period of time due to the sustained oil release and this property has been preserved in topical gel formulation.

**Conclusion**.

According the acquired results, SLN carrier can strongly potentiate the antibacterial effects of Lav-O leading to decrease the MIC from 0.12 mgml^− 1^ for Lav-O to 0.05 mgml^− 1^ in SLN form and when the nanoparticles were incorporated in topical gel formulations, they preserved their antibacterial properties (MIC: mgml^− 1^ mgml^− 1^). Hence, cholesterol and lecithin were the main lipids in this SLN structure, their natural and dermal compatible characteristics values its dermal penetration ability in nano-scale. Therefore, based on inherent wound healing properties of Lav-O and its potentiated antibacterial effects, a topical gel containing Lav-SLN could be a promising dosage form for wound healing which should be considered for future *in-vivo* and clinical evaluations.

## Data Availability

All data materials have been included in paper manuscript.
